# Endothelial Colony Forming Cells as an Autologous Model to Study Endothelial Dysfunction in Patients with a Bicuspid Aortic Valve

**DOI:** 10.3390/ijms20133251

**Published:** 2019-07-02

**Authors:** Vera van de Pol, Lidia R. Bons, Kirsten Lodder, Konda Babu Kurakula, Gonzalo Sanchez-Duffhues, Hans-Marc J. Siebelink, Jolien W. Roos-Hesselink, Marco C. DeRuiter, Marie-José Goumans

**Affiliations:** 1 Department of Cell and Chemical Biology, Leiden University Medical Center, 2300 RC Leiden, The Netherlands; 2 Department of Cardiology, Erasmus Medical Center, 3000 CA Rotterdam, The Netherlands; 3 Department of Cardiology, Leiden University Medical Center, 2300 RC Leiden, The Netherlands; 4 Department of Anatomy and Embryology, Leiden University Medical Center, 2300 RC Leiden, The Netherlands

**Keywords:** endothelial colony forming cell, ECFC, blood outgrowth endothelial cell, BOEC, bicuspid aortic valve, BAV, aortic dilation, calcification, migration

## Abstract

Bicuspid aortic valve (BAV), the most common congenital heart defect, is associated with an increased prevalence of aortic dilation, aortic rupture and aortic valve calcification. Endothelial cells (ECs) play a major role in vessel wall integrity. Little is known regarding EC function in BAV patients due to lack of patient derived primary ECs. Endothelial colony forming cells (ECFCs) have been reported to be a valid surrogate model for several cardiovascular pathologies, thereby facilitating an in vitro system to assess patient-specific endothelial dysfunction. Therefore, the aim of this study was to investigate cellular functions in ECFCs isolated from BAV patients. Outgrowth and proliferation of ECFCs from patients with BAV (*n* = 34) and controls with a tricuspid aortic valve (TAV, *n* = 10) were determined and related to patient characteristics. Interestingly, we were only able to generate ECFCs from TAV and BAV patients without aortic dilation, and failed to isolate ECFC colonies from patients with a dilated aorta. Analyzing EC function showed that while proliferation, cell size and endothelial-to-mesenchymal transition were similar in TAV and BAV ECFCs, migration and the wound healing capacity of BAV ECFCs is significantly higher compared to TAV ECFCs. Furthermore, calcification is blunted in BAV compared to TAV ECFCs. Our results reveal ECs dysfunction in BAV patients and future research is required to unravel the underlying mechanisms and to further validate ECFCs as a patient-specific in vitro model for BAV.

## 1. Introduction

Bicuspid aortic valve (BAV) is the most common congenital heart defect, present in 1–2% of the adult population worldwide. While a normal aortic valve consists of three leaflets (tricuspid aortic valve, TAV), a BAV has only two free moveable/floating leaflets either with or without a raphe. BAV can occur sporadically or can be inherited, and mutations in e.g., *NOTCH1*, *TGFBR1* and *SMAD6*, have been reported to be associated with BAV [1,2]. Furthermore, there is a remarkable male preponderance of 3:1 in the total population. Patients with a BAV have an increased prevalence of dilation and even rupture of the ascending aorta, while aortic valve regurgitation or stenosis can also occur [3]. 

Aortic dilation can become a life-threatening situation manifested by destructive changes in the aortic architecture, caused, among other reasons, by dedifferentiation of contractile smooth muscle cells (SMCs) and elastic lamella fragmentation [4]. To understand the cellular and molecular mechanisms driving the pathogenesis in aortic dilation, research over recent decades has mainly focused on SMCs and the role of biochemical signals to steer their differentiation and extracellular matrix modulation within the media of the aorta [5]. Little is known about a possible role of the endothelium in aortic dilation in BAV patients. Recently, the endothelial cells (ECs) have become of increased interest in BAV pathology (reviewed in [6]). Interestingly, altered EC migration, EC function and endothelial-to-mesenchymal transition (EndoMT) have been described in BAV patients [7,8]. Furthermore, mutations in *ROBO4* have recently been identified in BAV patients, which was shown to impair the barrier function of the ECs and induce EndoMT [9]. 

It is extremely difficult to obtain primary ECs from ascending aorta to study endothelial function, especially when matched controls are needed for comparison. Furthermore, patient-derived aortic ECs are a heterogeneous, non-proliferative population of ECs, derived from end-stage disease material [10]. Therefore, circulating endothelial progenitor cells have become an important tool to study EC function in different cardiovascular diseases. 

There are 2 main types of circulating endothelial progenitor cells described; namely, endothelial progenitor cells (EPCs) and endothelial colony forming cells (ECFCs). While EPCs express some EC markers such as PECAM1, von Willebrand Factor and VE-cadherin, it is now well established that these cells are CD14^+^ circulating mononuclear cells, instead of true endothelial progenitors [11]. Previous studies have shown that the number of EPCs is reduced in BAV patients with or without aneurysms, when compared to TAV patients with or without aneurysms, respectively [12]. In addition, BAV patients with dysfunctional valves have reduced numbers of circulating EPCs when compared to BAV patients with a normal functioning valve [13]. Moreover, EPCs exhibit a decreased migratory capacity in BAV patients with dysfunctional valves [13]. ECFCs, also known as blood outgrowth endothelial cells (BOECs), are the real circulating endothelial progenitor cells. ECFCs can be isolated from amongst other peripheral blood and give rise to a cell population indistinguishable from mature ECs [11,14]. These cells are able to contribute to vessel formation in vivo and have a high proliferative potential [11,15]. ECFCs have been used as a proxy to study EC function in diseases such as pulmonary arterial hypertension (PAH), diabetes and ischemic heart disease [16,17,18,19]. For example, in PAH, it is reported that failure of ECFC outgrowth is associated with clinical worsening [20]. 

To date, there is no data available describing the function of ECFCs in BAV patients. Given the important role of EC function in vessel stability, in this study we aimed to investigate EC function in BAV patients. Because ECFCs resemble EC function very well and isolating ECs from aortic tissue is not feasible, studying these cells may provide a valuable insight into EC functioning in BAV patients. Therefore, we isolated ECFCs from BAV patients and participants with a TAV serving as healthy controls. The outgrowth and proliferation of ECFCs was quantified and related to patient characteristics. Moreover, migration and response to calcifying stimulation was assessed in the ECFCs. Our results demonstrate ECFC dysfunction in BAV patients compared to healthy TAV controls. We expect that this will encourage other researchers to further develop and characterize ECFCs as an in vitro model for BAV.

## 2. Results

### 2.1. No Successful Growth of ECFC Colonies Isolated from Patients with a Dilated Aorta

**Figure 1:**
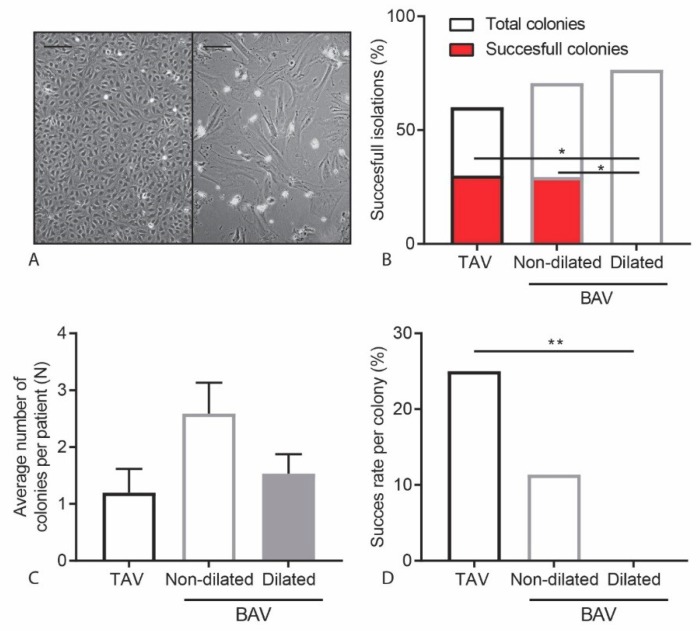
Successful growth of ECFCs in TAV and BAV non-dilated patients. (**A**) Representative images of a successful (left) and an unsuccessful (right) ECFC colony. Scalebar is 200 µm. (**B**) Graph showing the percentage of patient isolations resulting in a colony and the percentage of patient isolations resulting in a cell line. (**C**) Graph indicating the average number of colonies per isolation. (**D**) Graph showing the percentage of colonies resulting in a successful ECFC cell line. * *p* < 0.05, ** *p* < 0.01.

We first investigated whether ECFCs can be isolated from BAV patients and TAV controls. To isolate ECFC colonies, peripheral blood derived mononuclear cells were collected from patients (*n* = 34) and healthy participants (controls, *n* = 10). There were no significant differences between the included control participants and the patients with regard to age, height, weight and gender (Table 1). The isolated mononuclear cells fractions were seeded, and wells were monitored for colonies to appear after 2–5 weeks. In total, 74 colonies appeared, but not all colonies resulted in a successful ECFC patient-derived cell line. Growth of an ECFC colony was considered successful if they were able to proliferate for at least 8 passages. Unsuccessful ECFC isolations were those colonies that showed a decrease in proliferation rate, and adopted morphologically a senescent, mesenchymal phenotype (Figure 1A). 

There was no correlation between successful and unsuccessful ECFC isolations when comparing gender, age, length and weight between TAV control and BAV patients (Table 2). From the TAV control participants, 30% of the isolations lead to successful colony growth. For the isolations of BAV patients derived ECFCs there was a trend towards decrease in isolation efficiency with a success rate of 14.7% (χ2(1) = 1.22, *p* = 0.27). Interestingly, all isolations from BAV patients that did result in an ECFC cell line were isolated from BAV patients without aortic dilation (non-dilated BAV: nBAV, *n* = 17). We were unable to isolate stable ECFCs from BAV patients with aortic dilation (dilated BAV: dBAV, *n* = 17). The decreased isolation efficiency observed in dBAV patients was significantly different compared to both TAV control samples and nBAV patients (χ2(2)= 6.16, *p* = 0.046) (Figure 1B). When comparing patient characteristics, dBAV patients did have a significantly higher age and bodyweight (Table A1). Interestingly, the observed decrease in successful ECFC isolations was not due to a decrease in total number of colonies that appeared after seeding of the mononuclear cells (Figure 1B) or the number of colonies observed per patient (Figure 1C). There was a significant decrease in success per colony after the first passage in colonies from dBAV patients compared to colonies from TAV patients (χ2(1)= 7.075, *p* = 0.008) (Figure 1D). Our results demonstrate for the first time that the establishment of stable ECFCs cultures from BAV patients with an aortic dilation is compromised. 

There was no correlation between successful and unsuccessful ECFC isolations when comparing gender, age, length and weight between TAV control and BAV patients (Table 2). From the TAV control participants, 30% of the isolations lead to successful colony growth. For the isolations of BAV patients derived ECFCs there was a trend towards decrease in isolation efficiency with a success rate of 14.7% (χ2(1) = 1.22, *p* = 0.27). Interestingly, all isolations from BAV patients that did result in an ECFC cell line were isolated from BAV patients without aortic dilation (non-dilated BAV: nBAV, *n* = 17). We were unable to isolate stable ECFCs from BAV patients with aortic dilation (dilated BAV: dBAV, *n* = 17). The decreased isolation efficiency observed in dBAV patients was significantly different compared to both TAV control samples and nBAV patients (χ2(2) = 6.16, *p* = 0.046) (Figure 1B). When comparing patient characteristics, dBAV patients did have a significantly higher age and bodyweight (Table A1). Interestingly, the observed decrease in successful ECFC isolations was not due to a decrease in total number of colonies that appeared after seeding of the mononuclear cells (Figure 1B) or the number of colonies observed per patient (Figure 1C). There was a significant decrease in success per colony after the first passage in colonies from dBAV patients compared to colonies from TAV patients (χ2(1) = 7.075, *p* = 0.008) (Figure 1D). Our results demonstrate for the first time that the establishment of stable ECFCs cultures from BAV patients with an aortic dilation is compromised. 

### 2.2. EndoMT Response is Similar in TAV and BAV ECFCs

**Figure A1:**
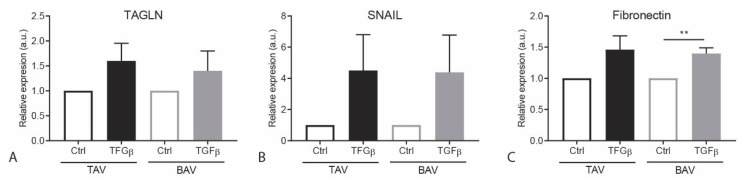
EndoMT related gene expression in TAV and BAV ECFCs upon TGFβ stimulation. Graphs showing the relative increase in expression upon TGFβ stimulation compared to not stimulated cells of (**A**) *TAGLN*, (**B**) *SNAIL* and (**C**) *FN1*. ** *p* < 0.01.

Recent studies demonstrate that EndoMT is altered in BAV patients compared to TAV controls [7,9,22,23]. To investigate this, we examined the ability of ECFCs to undergo EndoMT in response to TGFβ stimulation. After 48 h of TGFβ stimulation, we determined the expression levels of the EndoMT associated genes *SNAI1* (encoding Snail), *TAGLN* (encoding SM22a) and *FN1* (encoding Fibronectin) by qRT-PCR. As expected, an increase in mRNA expression of these EndoMT target genes was observed in both TAV and BAV ECFCs upon TGFβ stimulation. There was no difference TGFβ induced EndoMT between the TAV and BAV ECFCs (Figure A1).

### 2.3. Proliferation and Cell Size Are Similar in TAV and BAV ECFCs

**Figure 2:**
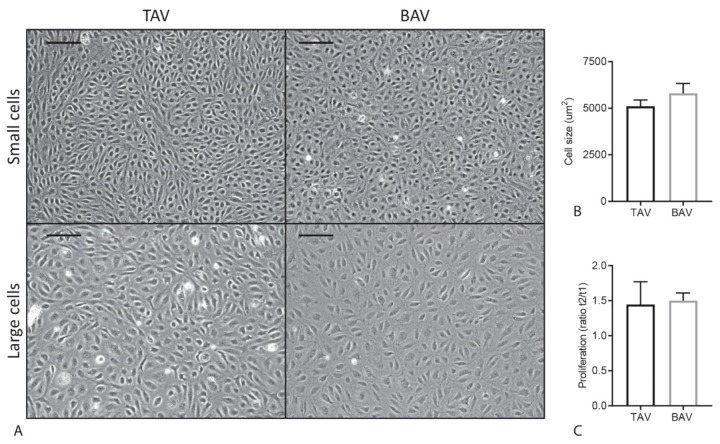
ECFC proliferation rate and cell size in TAV and BAV. (**A**) Representative images of variation in cell size in TAV and BAV ECFCs. The scale bar is 200 µm. (**B**) Graph showing TAV and BAV ECFC cell size. (**C**) Graph indicating TAV and BAV ECFC proliferation.

During cell culture, we observed an apparent variation in cell size and proliferation between the different ECFC cell lines (Figure 2A). These differences were not depending on confluence of the wells since they were observed in both confluent and non-confluent situations. Next, cell surface area was determined to investigate a potential relationship between cell size and aortic valve morphology, but no significant difference in cell size was found between TAV and BAV ECFCs (*p* = 0.9387) (Figure 2B). To determine if the variation in cell size is caused by changes in cell growth, cell proliferation rate was measured and depicted as the difference in cell number 24 and 72 h after seeding. As can be appreciated in Figure 2C, no difference in proliferation rate between TAV and BAV ECFCs was observed (*p* = 0.8407). In addition, there was no correlation between cell proliferation and cell size when comparing average proliferation rate and cell size per ECFC line (*p* = 0.321, data not shown). Furthermore, since during epithelial-to-mesenchymal transition (EMT) an increase in size of epithelial cells in transition is observed [24], we next analyzed if there was a correlation between cell size and EndoMT gene transcription, but no significant differences were observed (data not shown).

### 2.4. BAV ECFCs Migrate Faster Compared to TAV ECFCs

**Figure 3:**
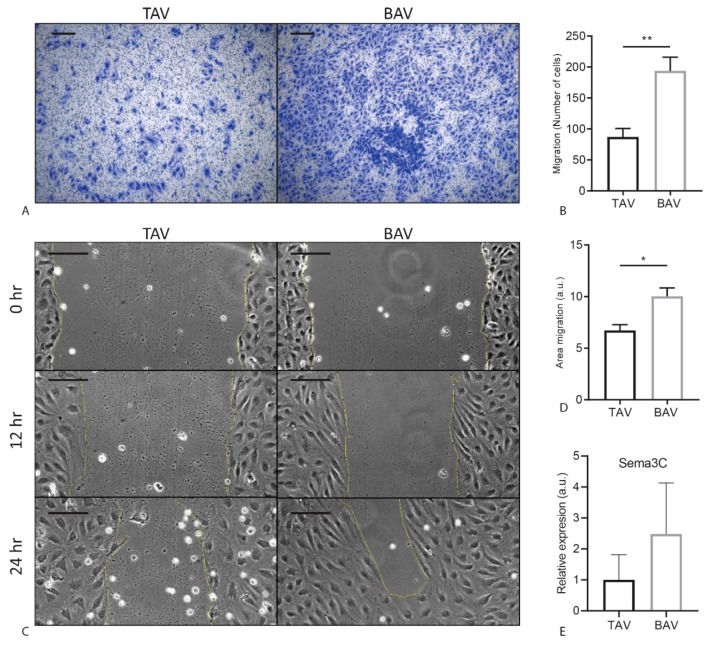
Migration assay of TAV and BAV ECFCs. (**A**) Representative images of TAV and BAV ECFC transwell migration stained with crystal violet. (**B**) Graph indicating TAV and BAV transwell migration, quantified by counting the number of ECFCs migrated in 24 hr. (**C**) Representative images of three different timepoints in the scratch assay of TAV and BAV ECFCs. (**D**) Graph showing TAV and BAV scratch migration quantification. Difference in area between 8 h and 12 h was measured. (**E**) Graph indicating qPCR results for *Sema3C*. Scalebar is 200 µm. * *p* < 0.05 ** *p* < 0.01, a.u.= arbitrary units.

Previous studies have shown that poor cell migration of EPCs correlated with worse valvular function in BAV [13]. Therefore, we next determined migration of ECFCs using two different assays, transwell and scratch assay. In order to assess the intrinsic migratory potential of the ECFCs, we first performed a transwell assay using serum as a migratory stimulus, and visualized the migrated cells using crystal violet (Figure 3A). There was a remarkable significant increase in migration of BAV ECFCs compared to TAV ECFCs (TAV: M = 87.2 SEM = 13.6, BAV: M = 193.9 SEM = 22.0, *p* = 0.0029) (Figure 3B). Next we performed a scratch (wound healing) assay. To determine the wound healing response, we calculated the difference in surface area between 2 timepoints. The scratch assay confirmed the difference observed in the transwells. There was a significant increase in wound closure by BAV ECFCs compared to TAV ECFCs (TAV: M = 6.73 SEM = 0.56, BAV: M = 10.03 SEM = 0.82, *p* = 0.020) (Figure 3C,D). Sempahorin3C (sema3C), a secreted guidance protein, regulates endothelial migration [25]. We therefore quantified *Sema3c* expression by qRT-PCR in TAV and BAV ECFCs, and observed a trend towards an increase in *Sema3C* expression in BAV ECFCs compared to TAV ECFCs (Figure 3E). Taken together, we show that migration of cells is reduced in BAV ECFCs. 

### 2.5. Calcification Is Decreased in BAV ECFCs Compared to TAV ECFCs

**Figure 4:**
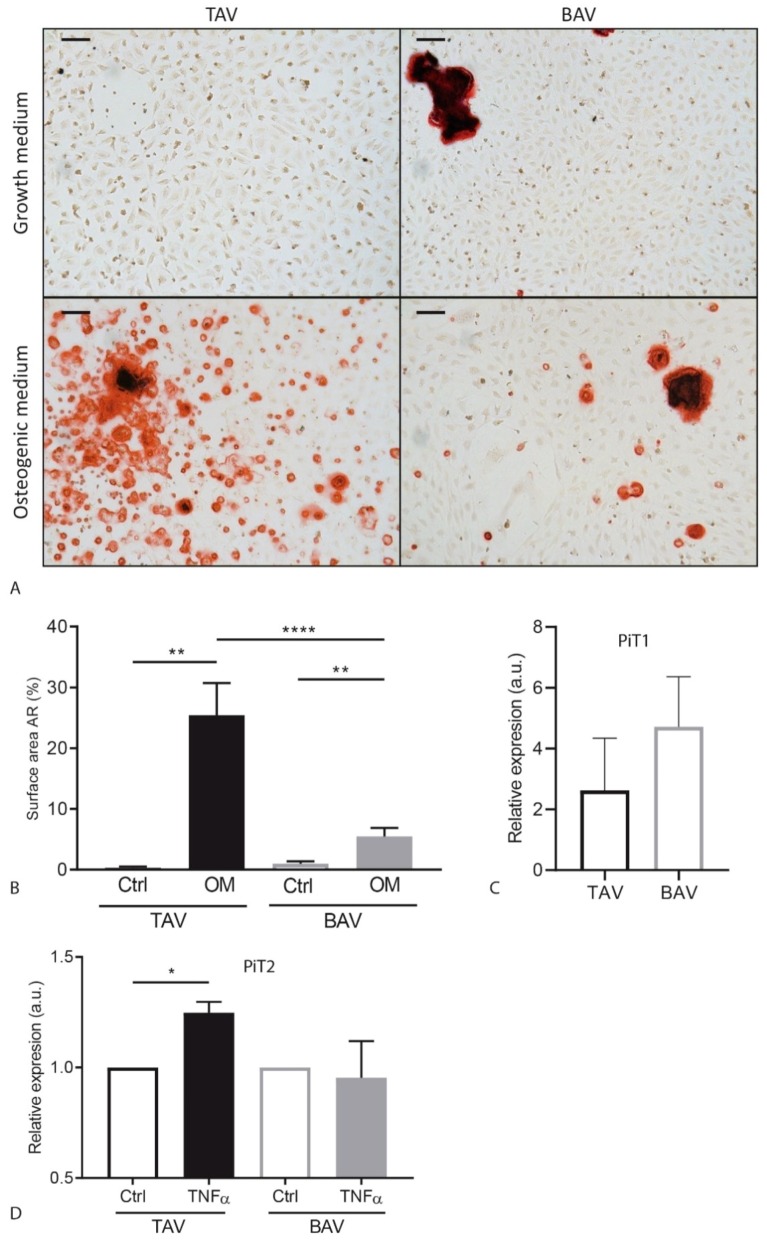
TAV and BAV ECFC calcification results. (**A**) Representative images of an Alizarin Red staining of TAV and BAV ECFCs after 18 days of culture in growth medium or osteogenic medium. (**B**) Graph of TAV and BAV calcification measured using picture analysis measuring surface area of Alizarin Red. (**C**) Graph indicating *PiT1* gene expression in BAV and TAV ECFCs under normal culture conditions. (**D**) Graph showing *PiT2* gene expression levels upon ECFC stimulation with TNFα. Scalebar is 100 µm. * *p* < 0.05 ** *p* < 0.01 **** *p* < 0.001.

**Figure A2:**
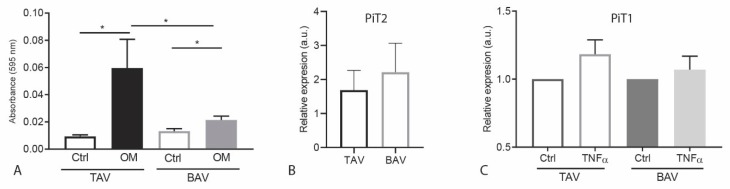
Calcification results of TAV and BAV ECFCs. (**A**) Graph of TAV and BAV calcification measured using absorbance after dissolving alizarin red. (**B**) Graph indicating *Pi2* gene expression in BAV and TAV ECFCs under normal culture conditions. (**C**) Graph showing *PiT1* gene expression levels upon ECFC stimulation with TNFα. * *p* < 0.05.

Since valvular calcification is a serious complication in BAV patients, we studied the ability of the BAV and TAV ECFCs to calcify. After culturing ECFCs in osteogenic medium we stained the wells with Alizarin Red (Figure 4A). Interestingly, calcium deposits could already be detected in some of the samples after being cultured for 18 days in growth medium. These deposits were more present in the BAV than the TAV ECFCs; however, the difference was not significant (TAV: M = 0.35% SEM = 0.24, BAV: M = 0.97% SEM = 0.41, *p* = 0.31) (Figure 4A,B, Figure A2a). Culturing the cells osteogenic medium increased the amount of Alizarin Red staining in the TAV and BAV ECFCs when compared to growth medium. Although both showed an increase in calcium deposition, this increase is less in BAV ECFCs when compared to TAV ECFCs when cultured in osteogenic medium (Figure 4A,B, Figure A2a). 

Previously it has been shown that inorganic phosphate transporter 1 and 2 (PiT1 and PiT2) are expressed in progenitor cells during differentiation towards osteoblasts [26]. Furthermore, an increased expression of *PiT1* is related to increased mineralization and shown to be involved in SMC transdifferentiation into osteoblast-like cells [27,28]. Therefore, we determined whether PiT1 and PiT2 may play a role in ECFC calcification and found a trend towards increased expression of both *PiT1* and *PiT2* in BAV ECFCs compared to TAV ECFCs under normal culture conditions (Figure 4C, Figure A2b). 

Inflammatory stimulation using tumor necrosis factor (TNF)-α has been reported to sensitize ECFCs for calcification [29]. Therefore, we stimulated ECFCs with TNFα for 48 h and determined the expression levels of *PiT1* and *PiT2*. Interestingly, *PiT2* expression upon TNFα significantly increased in TAV ECFCs (*p* = 0.0157, 24.8%, SEM = 0.05) but not in BAV ECFCs (*p* = 0.788, −4.5%, SEM = 0.166) (Figure 4D). In addition, the expression of *PiT1* showed a similar trend (Figure A2c). In summary, ECFCs from BAV patients exhibit a reduced capacity to calcify when compared to TAV derived ECFCs and TNF-α stimulation specifically induced the expression of *PiT2* in TAV ECFCs, but not in BAV ECFCs.

## 3. Discussion

In this study, we isolated ECFCs from BAV patients and TAV controls to study EC function, and observed a striking decrease in efficiency of successful ECFC isolation in BAV patients with a dilated aorta. In the ECFCs that were successfully isolated, proliferation rate and cell size were similar, but there was a decrease in migratory behavior of ECFCs from BAV patients when compared to TAV patients. While no differences were detected in their response to TGFβ induced EndoMT, we did observe a reduced calcification response in BAV ECFCs compared to TAV ECFCs. 

Normal endothelial function is pivotal for a healthy cardiovascular function. In the past years, research to understand bicuspid aortic valve disease has suggested that endothelial dysfunction might play a role [7,9,30]. In vitro characterization of ECs isolated from aneurysmal aortic tissue show a decreased proliferation rate in BAV aortic ECs compared to TAV control and TAV aneurysmal ECs [31]. Unfortunately, primary ECs isolated from aortic tissue are heterogeneous, have very limited proliferative capacity and can only be obtained from end stage disease surgical material, hampering progress in our understanding of the impact of the endothelium in BAV. Therefore, we took the approach to generate ECFC cell lines from BAV patients to study EC function. ECFCs not only provide an in vitro cell model for patient specific endothelial functioning, but in vivo they contribute to endothelial wound healing [14,15,16]. Therefore, altered function of ECFCs might contribute to endothelial dysfunction when e.g., repairing the endothelial layer damaged due to years of altered wall shear stress [32]. Although the behavior of ECFCs might not fully recapitulate the ECs present in the aortic wall, these cells cannot be obtained from healthy matched controls. ECFCs are an EC source that most closely resemble mature ECs obtained using a minimal invasive isolation protocol [11,14]. Peripheral ECs can also be isolated to study patient specific EC functioning, but they have been reported to have clear different characteristics from aortic ECs, and would cause a higher isolation burden. Therefore, we decided to use ECFCs to study patient specific EC function [33].

The isolation efficiency of ECFCs from BAV patients with a non-dilated aorta was similar to TAV control. Interestingly, we were unable to isolate ECFCs from BAV patients with an aortic dilation. This observation might suggest a possible biomarker role of ECFCs in BAV aortic dilation. A recent study analyzing EPCs reported a reduced number of EPCs in BAV patients with a dysfunctional aortic valve when compared to a normal functioning BAV [13]. A decrease in circulating progenitor cells in BAV patients with additional cardiovascular pathologies could explain the lack of successful ECFC isolations from dBAV patients. Incongruently, we were able to successfully isolate ECFCs from patients with valvular dysfunction. Moreover, we did not observe a difference between the number of colonies that appeared in isolations from BAV patients with or without aortic dilation compared to TAV ECFCs. We did observe a difference in the proliferative capacity of the different colonies. All colonies isolated from dBAV patients stopped proliferating soon after appearing and gained a senescent/mesenchymal morphology. Recent studies have shown that there was an increase in EndoMT gene expression profile associated with a BAV [7,9,22,23]. Increased EndoMT could be the cause of an increased amount of BAV colonies to gain a mesenchymal phenotype. Moreover, as epithelial cell size has been correlated to EMT, the different cell sizes observed in the ECFC culture could be related to an EndoMT phenotype [24]. However, in the BAV ECFCs we did not observe a difference in upregulation of expression of EndoMT related genes upon TGFβ stimulation between TAV and BAV ECFC cell lines. Moreover, we did not observe a correlation between cell size and the expression of different EndoMT related target genes. Although increased age has been reported to impair ECFC isolation [34], but we did not observe a significant age difference when comparing successful and non-successful isolations in this study. There was, however, a significant increase in age of patients with a dilated aorta compared to BAV patients without a dilated aorta. 

To study EC behavior, migration of the ECFCs was investigated. Our data shows that migration is increased in BAV ECFCs when compared to TAV ECFCs. Moreover, BAV ECFCs show an increase in *Sema3C* expression, a secreted protein that regulates EC function and enhances migration. Reduced EC migration in vitro has been found in many different diseases, e.g., in ECFCs derived from patients with diabetes or preeclampsia [35,36], and related to the functionality of the patient specific ECs. In contrast to diabetes and preeclampsia, we observed an increase in migration of ECFCs derived from BAV patients, leaving us to speculate how this affects the function *in vivo*. Migratory behavior has been studied using aortic SMCs from BAV patients with an aortic aneurysm. When comparing them to TAV non-aneurysmal aortic SMCs, these BAV SMCs show a decreased migration rate [31]. Either no difference, or reduced migration has been observed in SMC migration from BAV aneurysmal aorta when compared to TAV aneurysmal aorta. Since no SMCs were isolated from BAV with a non-dilated aorta it is difficult to draw any conclusion if SMC migration in BAV patients is already altered prior to vessel dilation. Finally, a decrease in migration of BAV EPCs was related to increased dysfunction in aortic valves [13], but unfortunately our study is not sufficiently powered to be able to relate patient characteristics to aortic valve dysfunction. 

Because valvular calcification is a common problem in a subpopulation of patients with a BAV, we studied calcification of the ECFCs. Interestingly, BAV ECFCs cultured in a well for 18 days on growth medium show more calcium deposits compared to TAV. Moreover, expression of *PiT1*, a gene shown to be involved in calcification, shows a modest increase in BAV ECFCs [26,27,28]. Since patients with a BAV have a high chance to develop calcific aortic valve disease (CAVD), we expected BAV ECFCs to calcify more than TAV ECFCs. However, upon osteogenic stimulation, BAV ECFCs showed a much smaller increase in calcium deposition than TAV ECFCs did. This unexpected result might be explained by differences between in vitro and in vivo cell function, and/or the calcifying stimulation in vivo in BAV is different from our experimental set-up. It does, however, confirm that the calcification response in BAV ECFCs is altered compared to TAV ECFCs. Inflammatory stimulation using TNFα has been shown to sensitize ECFCs for calcification [29]. Moreover, a recent study on a large CAVD cohort showed that inflammatory markers were increased in CAVD BAV patients compared to CAVD TAV patients [37]. Therefore, we stimulated the ECFCs with TNFα, which caused significant increase in mRNA expression of *PiT2* in TAV ECFCs but not in BAV ECFCs. This lack of increase in *PiT2* could explain the reduced response of BAV ECFCs to osteogenic medium. 

In this study we have for the first time characterized ECFCs from BAV patients. Our data suggests that EC dysfunction is present in these patients, and future research should focus on the role of endothelial dysfunction in the pathogenesis of BAV and the related aortic valve calcification and aortic dilation, taking into account the role of BAV related genes that are known to affect cell migration, proliferation, EndoMT and calcification such as *SMAD6*, *NOTCH1* or *TGFβR1* [1,2] [38,39,40,41]. Altogether, we expect that this study will stimulate the use of ECFCs as a surrogate model to determine endothelial function in BAV.

## 4. Materials and Methods 

Ethical approval for this study was obtained from the Medical Ethical Committee from the Leiden University Medical Center (METC LUMC), Leiden, The Netherlands (P15.377, approved on 02-June-2016). The included patients were 18 years of age or older, had a bicuspid aortic valve and were invited to participate during routine visit to the outpatient clinic. Patients were excluded if they had undergone aortic valve surgery or intervention. Control participants were age and gender matched to the average of the patient population and a TAV was confirmed. The ascending aortic diameter was measured with echocardiography using the leading edge-to-leading edge technique [42]. An ascending thoracic aortic diameter equal to or larger than 40 mm was considered dilated. After obtaining written informed consent, peripheral blood (60 mL) was drawn from BAV patients and TAV control participants. To isolate the mononuclear cell fraction, the blood was diluted 1:1 with PBS and centrifuged using a ficoll gradient. In detail, the sample was divided in fractions of 25 mL which were gently pipetted in a 50 mL tube on top of 12.5 mL Ficoll Paque Plus (GE Healthcare, 17-1440-03, Chicago, Il, USA). The tubes were centrifuged at 750 G for 30 min without brake. The plasma layer was aspirated, and the buffy coat of the mononuclear cell fraction was transferred to a new 50 mL tube. The cells were washed 3× with PBS supplemented with PenStrep (100 U/mL, Thermo Fischer Scientific, Waltham, MA, USA) by adding 25 mL PBS to the tube, centrifuging 5 min at 230 G and aspirating the supernatant. The pellet was resuspended in 12.5 mL growth medium (EGM-2 (Lonza, CC-3162, Basel Switzerland) supplemented with 8% extra fetal bovine serum (FBS) and PenStrep (100 U/mL, Gibco). 48-Well plates were coated with 50 µg/mL purified bovine collagen (Advanced Biomatrix, #5005, Carlsbad, CA, USA) in MQ, 250 µL per well and were incubated 2 h at 37 °C and washed 3× using PBS before plating the resuspended cells. The medium was replaced for the first time after 3 days using growth medium, after which the medium was replaced 2 times per week. Colonies that appeared 2 to 5 weeks after isolation were passaged when covering approximately 25% of the well. Cells were dissociated by incubating the ECFCs for 1 min with EDTA (0.5 mM, Sigma, Saint Louis, MO, USA) followed by incubation with Trypsin 0.25%/EDTA (1:1 Serva, Heidelberg, Germany) for approximately 5 min until cells were dissociated from the well. This dissociation was confirmed using a microscope, after which they were resuspended and reseeded 1:2 onto collagen coated wells. The ECFCs were checked for expression of PECAM1 (qRT-PCR and Western blot) and VE-Cadherin (qRT-PCR) expression and absence of the lymphocyte marker CD45 was confirmed.

### 4.1. Protein Isolation and Western Blot

To isolate protein, ECFCs were grown to confluence, washed with PBS and lysed in Giordano buffer (50 nM Tris-HCl (pH7.4), 250 mM NaCl, 0.1% Triton X-100 and 5 mM EDTA) with 15% glycerol and protease inhibitors. Protein concentration was determined using Bradford Reagent (Bio-Rad, 500-0006, Hercules, CA, USA). Equal amounts of protein were loaded and separated by SDS-PAGE and transferred to Immobilon-P PVDF membranes (Merck, IPVH00010, Darmstadt, Germany). Membranes were blocked in Tris-buffered saline, 0.2% Tween-20 (TBST) containing 10% dry milk and incubated overnight with Pecam1 antibody (Santa Cruz Biotechnology, sc-1506-r, clone M-20, Dallas, TX, USA) diluted 1:1000 in 5% BSA in TBST. Membranes were washed 3 times with TBST and incubated with a horseradish peroxidase-conjugated goat-anti-rabbit antibody (Thermo Fischer Scientific, 31458) diluted 1:10,000 in 10% dry milk in TBST. Membranes were washed with TBST again, after which protein expression was detected using enhanced chemiluminescence (WesternBright Quantum, Advansta, San Jose, CA, USA) and visualized on x-ray film (Fuji film, Minato, Tokia, Japan).

### 4.2. mRNA Isolation and Quantitative RT-PCR

RNA was isolated using the ReliaPrep RNA cell miniprep kit (Promega, Z6012, Madison, WI. USA) according to the manufacturer’s protocol. RevertAid First Strand cDNA Synthesis (ThermoFisher Scientific, K1622) was used to generate cDNA according to the manufacturer’s protocol, after which qRT-PCR was performed using GoTaq qPCR Master Mix (Promega, A6001). *GAPDH* and *ARP* were used as housekeeping genes. Primer sequences used are detailed in the Supplementary Materials. 

### 4.3. Cell Size

To measure cell size, pictures were acquired from confluent areas. In these pictures, the number of cells was determined in an area of 2 mm^2^ from which an average cell surface area was calculated. Then the average cell surface area was calculated (2 mm^2^/number of cells).

### 4.4. Proliferation

Cell proliferation was determined by calculating the increase in cell number in 48 h. To this end, 15,000 cells were seeded per cm^2^ in growth medium which did not reach confluency before the end of the experiment. To quantify proliferation, cells were dissociated to single cells 24 and 72 h after seeding and automatically counted 3 times using a TC20 automated cell counter (Bio-Rad). The ratio between the number of cells of day 3 and day 1 was used as an indication for proliferation rate. 

### 4.5. MTT and PrestoBlue Assays

MTT and PrestoBlue assays were used to determine proliferation rate. For both assays 3000 cells/well were seeded in 96-well plates in a final volume of 100 μL/well. For the MTT assay, the growth medium was replaced by growth medium supplemented with 0.5 mg/mL 3-(4,5-Dimethylthiazol-2-yl)-2,5-diphenyltetrazolium bromide (MTT, Sigma M5655) for 3 hours at 37 °C, 16 (t1) and 40 h (t2) after seeding. The absorbance was measured at 595 nm, as described previously [43]. For the PrestoBlue assay, the growth medium was replaced with growth medium supplemented with 4% PrestoBlue Cell Viability Reagent (ThermoFisher, A13261). After 3 h of incubation at 37 °C, fluorescence was measured at 590 nm as a read out for mitochondrial respiration on a victor3V multilabel reader (Perkin Elmer, Waltham, MA, USA). Three technical replicates were included in each experiment and assays were repeated at least twice.

### 4.6. EndoMT Assay

To induce EndoMT, growth medium supplemented with 5 ng/mL TGFβ3 was added to a 6-wells well of ECFCs with a confluency of 60%. After 48 h, the cells were washed with PBS and RNA was isolated as described above. 

### 4.7. Transwell Migration

To measure migration towards a stimulus, a transwell migration assay was performed. A transwell insert with 5µm pores (Corning, CLS3421-48EA, Corning, NY, USA) was coated using 50 µg/mL bovine-collagen. Next, 10,000 ECFCs in 100 µL EGM2 (2%FBS) were seeded into the transwell insert. The bottom well contained 600 µL growth medium (10% FBS). After 24 h, the inserts were washed with PBS and fixated for 10 min with 4% paraformaldehyde (PFA) before staining the membrane with 4% Crystal violet (Sigma, C0775) in methanol. The ECFCs that did not migrate through the membrane were removed before taking pictures. Migrated ECFCs were counted from the pictures taken (5 images per transwell). 

### 4.8. Scratch Migration

A scratch assay was performed as an in vitro model for wound healing. The scratch assay was performed in a 24 well with a confluent layer of cells. Cells were scraped using a 200 µL tip after which they were automatically imaged for 24 h. To calculate the migration rate, the open area was measured at 8 and 12 h after making the scratch. The difference in area between the timepoints was determined to calculate the migration speed. 

### 4.9. Calcification Assay

To study calcification, ECFCs were seeded in triplo in 48-wells plates. When reaching confluency, the ECFCs were incubated with osteogenic medium (growth medium supplemented with 2.7 mM CaCl_2_ and 2.5 mM NaH_2_PO_4_) which was replaced twice-weekly. After 18 days, the ECFCs were fixated using 4% PFA for 10 min, washed with MQ and incubated with 2% Alizarin Red (Sigma, TMS-008-C, pH 4.2) in MQ for 3 min. The plates were washed again 2× with MQ before imaging. Surface area of AR in the images was automatically measured using Fiji [44]. Finally, the alizarin red was dissolved by replacing the MQ with 150 µL cetylperidiumchloride for 3 h at 37 °C, after which the absorbance was measured in duplo at 595 nm. To study the effect of inflammation on calcification, ECFCs were stimulated with 10 ng/mL TNFα (Peprotech, 300-01A, Rocky Hill, NJ, USA) for 48 h before isolating RNA, as described above, and studying gene expression by qRT-PCR. 

### 4.10. Statistics

All results were obtained in at least 4 independent experiments. The isolation and initial culture, as well as the first round of experiments were performed while blinded. Statistical assays were performed using Graph Pad Prism (version 7). Correlations were tested using Pearson correlation. All other statistical tests were performed using Students t-test, except from the number of colonies per patient, which was analyzed using Fischer’s exact test and one-way ANOVA for the % of successful colony growth. qRT-PCR results from the experiments with TNFα and TGFβ stimulation are related to the non-stimulated control per cell line. *p*-values < 0.05 (*), 0.01 (**), 0.001(****) were considered significantly different.

## Figures and Tables

**Table 1:** Participant characteristics.

	TAV (10)	BAV (34)	
**Male Sex, *n* (%)**	5 (50)	18 (52.9)	
**Age, Year (stdev)**	32.0 (10.5)	38.4 (15.5)	
**Height, cm (stdev)**	179.4 (15.8)	176 (11.0)	
**Weight, kg (stdev)**	74.8 (11.2)	76.9 (14.5)	
**Valve Types, *n* (%) ^1^**			
Type 0	-	5 (14.7)	
Type 1 L-R	-	17 (50)	
Type 1 R-N	-	4 (11.8)	
Type 2 L-R, R-N	-	2 (5.9)	
Unknown	-	6 (17.6)	
**Dilation, Yes (%)**	0 (0)	17 (50) *	
**Aortic Size, mm (stdev)**	all	non-dilated	dilated
Aorta ascendens	28.6 (3.0)	30.2 (3.0)	43.1 (5.5)
**Stenosis, *n* (%)**			
Severe	0 (0)	4 (11.8)	
Moderate	0 (0)	6 (17.6)	
Mild	0 (0)	15 (14.7)	
None	10 (100)	19 (55.9)	
**Insufficiency, *n* (%)**			
Moderate	0 (0)	10 (29.4)	
Mild	0 (0)	12 (35.3)	
None	10 (100)	17 (50)	

^1^ Valve classification according to Sievers [21].

**Table 2:** Participant characteristics successful ECFC isolations.

	TAV (3, 30.0%)	BAV (5, 14.7%)	
**Male Sex, *n* (%)**	2 (66.7)	3 (60)	
**Age, Year (stdev)**	34.0 (12.2)	25.8 (5.9)	
**Height, cm (stdev)**	186.3 (5.1)	174.6 (14.0)	
**Weight, kg (stdev)**	77.7 (10.0)	66.4 (10.3)	
**Valve Types, *n* (%)**			
Type 0	-	2 (20)	
Type 1 L-R	-	2 (20)	
Type 1 R-N	-	0 (0)	
Type 2 L-R, R-N	-	0 (0)	
Unknown	-	1 (20)	
**Dilation, Yes (%)**	0 (0)	0 (0) *	
**Aorta Ascendens Size, mm (stdev)**	28 (2.0)	29.8 (2.3)	
**Stenosis, *n* (%)**			
Severe	0 (0)	0 (0)	
Moderate	0 (0)	1 (20)	
Mild	0 (0)	1 (20)	
None	3 (100)	3 (60)	
**Insufficiency, *n* (%)**			
Moderate	0 (0)	1 (20)	
Mild	0 (0)	1 (20)	
None	3 (100)	3 (60)	

## Supplementary Materials

Supplementary materials can be found at https://www.mdpi.com/1422-0067/20/13/3251/s1.

## Abbreviations

BAV	Bicuspid aortic valve
CAVD	Calcific aortic valve disease
ECFC	Endothelial Colony Forming Cells
EC	Endothelial cell
EPC	Endothelial Progenitor Cells
EndoMT	Endothelial-to-Mesenchymal transition
FBS	Fetal Bovine Serum
TAV	Tricuspid aortic valve
TGFβ	Transforming growth factor β
TNFα	Tumor necrosis factor α

## Appendix A

**Table A1:** BAV patient characteristics with/without aortic dilation.

	Non-Dilated Aorta (17)	Dilated Aorta (17)
**Male Sex**, *n* (%)	7 (41.2)	11 (64.7)
**Age,** year (stdev)	28.9 (7.8)	47.9 (15.7) ^#^
**Height**, cm (stdev)	173.9 (11.8)	178.4 (9.9)
**Weight**, kg (stdev)	69.5 (12.5)	84.2 (12.9) **
**Valve Types**, *n* (%)		
Type 0	4 (23.5)	1 (5.9)
Type 1 L-R	8 (47.1)	9 (52.9)
Type 1 R-N	1 (5.9)	3 (17.6)
Type 2 L-R, R-N	1 (5.9)	1 (5.9)
Unknown	3 (17.6)	3 (17.6)
**Aortic Size**, mm (stdev)	30.2 (3.0)	43.1 (5.5)
**Stenosis**, *n* (%)		
Severe	2 (11.8)	1 (5.9)
Moderate	3 (17.6)	4 (23.5)
Mild	2 (11.8)	3 (17.6)
None	10 (58.8)	9 (52.9)
**Insufficiency**, *n* (%)		
Moderate	2 (11.8)	7 (41.2)
Mild	3 (17.6)	4 (23.5)
Trace	4 (23.5)	2 (11.8)
None	8 (47.1)	4 (23.5)

** *p* < 0.01, # = *p*<0.0001.
