# Supplementary material for: Endothelial Colony Forming Cells as an Autologous Model to Study Endothelial Dysfunction in Patients with a Bicuspid Aortic Valve
Source: Int J Mol Sci. 2019 Jul 2;20(13):3251. doi: 10.3390/ijms20133251 (PMC6651394; doi:10.3390/ijms20133251)
Supplement: Supplementary file 1 [file ijms-20-03251-s001.pdf]

Supplementary Materials: Primer sequences for qPCR.

gene	forward*	reverse*
GAPDH	AGCCACATCGCTCAGACAC	GCCCAATACGACCAAATCC
ARP	CACCATTGAAATCCTGAGTGATGT	TGACCAGCCGAAAGGAGAAG
PECAM1	ATCGGTTGTTCAATGCGTCC	CCTTCAGGATTTGGTACATGACA
VE-Cadherin	CTGCATCCTCACCATCACAG	ACCGACACATCGTAGCTGGT
Transgelin	TTCAAGCAGATGGAGCAGGT	TGCCATGTCTTTGCCTTCAA
Fibronectin	CGTCATAGTGGAGGCACTGA	CAGACATTCGTTCCCACTCA
SNAI1	GAGGACAGTGGGAAAGGCTC	TGGCTTCGGATGTGCATCTT
PiT1	GTTCGTGCATTCATCCTCCAT	TGGTACCCCACAGAGGAAGTTT
PiT2	TCTCATGGCTGGGGAAGTTAGT	TTGCGACCAGTGAGAATCCTAT
CD45	ATAGTCTGCCACGCCTCTG	AGTGTGAAGCGGCCAATG

* 5′end to 3′end.
